# Lysophosphatidic Acid 18:0 sn-1 in Cerebrospinal Fluid as a Potential Biomarker of Depressive Symptoms in Patients with Neuropathic Pain

**DOI:** 10.3390/brainsci16060573

**Published:** 2026-05-28

**Authors:** Reo Inoue, Masahiko Sumitani, Atsushi Kimura, Takao Mochizuki, Toru Akiyama, Yoshifumi Morita, Takuya Takahashi, Takashi Hirai, Kentaro Hayakawa, Hirotaka Chikuda, Makoto Kurano

**Affiliations:** 1 Department of Anesthesiology and Pain Relief Center, The University of Tokyo Hospital, Bunkyo 113-0033, Japan; inouer-ane@h.u-tokyo.ac.jp; 2 Department of Pain and Palliative Medicine, The University of Tokyo Hospital, Bunkyo 113-0033, Japan; 3 Department of Orthopaedic Surgery, Jichi Medical University, Shimotsuke 329-0498, Japan; 4 Department of Orthopaedic Surgery, Saitama Medical Center, Jichi Medical University, Omiya 330-0834, Japan; 5 Department of Clinical Laboratory Medicine, The University of Tokyo Hospital, Bunkyo 113-0033, Japan; moritay-lab@h.u-tokyo.ac.jp (Y.M.);; 6 Department of Orthopaedic and Spinal Surgery, Graduate School of Medical and Dental Sciences, Institute of Science Tokyo, Bunkyo 113-8519, Japanhirai.orth@tmd.ac.jp (T.H.); 7 Department of Orthopaedics and Spine Surgery, Tokyo Metropolitan Institute for Geriatrics and Gerontology, Itabashi 173-0015, Japan; 8 Department of Orthopaedic Surgery, Gunma University, Maebashi 371-8511, Japan

**Keywords:** lysophosphatidic acid, LPA, depressive symptoms, neuropathic pain, cerebrospinal fluid

## Abstract

**Highlights:**

**What are the main findings?**

- CSF LPA18:0 sn-1 correlated with depressive symptoms in neuropathic pain.
- LPA18:0 sn-1 remained independently associated after adjustment for pain-related factors.

**What are the implications?**

- LPA18:0 sn-1 may represent a candidate biomarker of depressive distress in neuropathic pain.
- Objective biomarkers may improve multidimensional pain assessment.

**Abstract:**

Background/Objectives: Lysophosphatidic acid (LPA) is a bioactive lipid mediator implicated in neuropathic pain (NP). Depressive symptoms frequently accompany NP and adversely affect outcomes, yet objective biomarkers remain limited. This study aimed to identify cerebrospinal fluid (CSF) LPA molecular species associated with depressive symptoms in patients with NP. Methods: CSF samples were obtained from 48 patients, and LPA species, including positional isomers (sn-1 and sn-2), were quantified using liquid chromatography–tandem mass spectrometry. Depressive symptoms were assessed using the depression subscale of the Hospital Anxiety and Depression Scale (HADS-D). Statistical analyses were performed to evaluate associations among depressive symptoms, clinical variables, and LPA species. Results: CSF LPA18:0 was significantly associated with HADS-D scores (r = 0.380, *p* = 0.010), with the strongest association observed for the sn-1 isoform (r = 0.385, *p* = 0.009). In multivariable analysis, LPA18:0 sn-1 remained independently associated with depressive symptoms, alongside pain intensity and pain catastrophizing (R^2^ = 0.386). Structural equation modeling supported an association between LPA18:0 sn-1 and depressive symptoms independent of pain-related factors. Conclusions: These findings suggest that CSF LPA18:0 sn-1 may be associated with a biological dimension of depressive distress and may represent a candidate biomarker of depressive distress in NP. However, these findings should be interpreted cautiously, as the analyses were exploratory, and further validation in independent and longitudinal cohorts is warranted.

## 1. Introduction

Lysophospholipids, derived from diacyl phospholipids, are key components of biological membranes. Lysophospholipids and phospholipids share a glycerol backbone; however, lysophospholipids contain one fatty acid chain, while phospholipids have two. Several lysophospholipids possess potent biological properties and are considered second-generation lipid mediators, similar to eicosanoids. One example is lysophosphatidic acid (LPA), a lysophospholipid consisting of a glycerol backbone with a phosphate group at the sn-3 position and a single fatty acyl chain at the sn-1 or sn-2 position (Figure S1). Additionally, lysophosphatidylcholine (LPC), a major precursor of LPA generated by autotaxin (ATX), is associated with the pathophysiology of neuropathic pain (NP). As LPC is a direct precursor of LPA, the LPC–LPA balance may reflect upstream lysophospholipid metabolism rather than downstream receptor signaling. Accordingly, the LPA/LPC ratio may provide complementary information regarding lysophospholipid dynamics in the central nervous system. In this study, LPC species and LPA/LPC ratios were analyzed as supplemental data to aid interpretation of LPA profiles, as the primary hypothesis focused on bioactive LPA species rather than precursor availability.

LPA species vary in their fatty acyl chain length and degree of saturation [1], resulting in species-dependent biological effects. LPA exerts various biological effects across multiple tissues and organs, including cellular proliferation, apoptosis inhibition, cytokine and chemokine secretion, platelet activation, and smooth muscle contraction [2]. LPA mediates its actions through specific G protein-coupled receptors, with six LPA receptors (LPA1–LPA6) identified to date [3]. In the nervous system, LPA is a key lipid mediator in the development and maintenance of NP [4]. Findings from basic NP model animals have been supported by clinical observations in patients experiencing NP [5,6,7].

NP causes multidimensional impairment and affects physical and mental well-being [8]. Patients with NP frequently experience severe sleep disturbances, difficulty concentrating, and fatigue. Additionally, many patients report experiencing depression and anxiety as significant sources of distress, even in the absence of a formal diagnosis of major depressive disorder [9]. Psychological symptoms and pain severity interact bidirectionally, often displaying a linear relationship [10]. However, in some cases, psychological distress may exacerbate pain severity in a nonlinear and potentially exponential way [10]. Patients with NP have physical health-related quality of life (QOL) scores comparable to those with severe physical illnesses, such as recent myocardial infarction, chronic heart failure, or poorly controlled diabetes. Furthermore, their mental health-related QOL is similarly reduced to levels observed in patients with major depressive disorder [11]. Given these findings, identifying biomarkers and understanding the mechanisms underlying psychological distress in patients with NP could offer valuable insights for improving assessment and treatment strategies.

Recent studies have associated LPA with neuropsychological functions and neuropsychiatric disorders. For instance, mice deficient in LPA receptors exhibit behavioral traits associated with anxiety and depression [12] or schizophrenia [13], indicating a potential link between LPA signaling and the pathophysiology of psychological distress. In this study, we investigated the associations between psychological symptoms and the cerebrospinal fluid (CSF) profiles of LPA species in patients with NP and constructed a structural model to examine their interrelationships.

However, objective biomarkers reflecting depressive symptoms in patients with NP remain limited, and their biological underpinnings are not fully understood.

## 2. Methods

This was a cross-sectional observational study conducted at a single tertiary care center to investigate the associations between cerebrospinal fluid LPA species and depressive symptoms in patients with neuropathic pain.

### 2.1. Participants

The study protocol was approved by the Institutional Review Board of the Ethics Committee of the University of Tokyo [approval number: 10516; firstly approval date: 1 March 2013, and updated 5-yearly (last updated: 22 February 2023)].

All procedures were conducted in accordance with the Declaration of Helsinki. Written informed consent was obtained from all participants prior to enrollment. The inclusion and exclusion criteria are described in detail elsewhere [5,6]. A certified orthopedic or pain physician diagnosed the participants with NP based on the criteria established by the International Association for the Study of Pain [14]. Patients with a medical history indicative of a disease affecting the peripheral and/or central somatosensory nervous system and pain exhibiting a neuroanatomically plausible distribution were diagnosed. The presence of the disease was confirmed using at least one diagnostic test, including a neurological examination, imaging, or electrophysiological studies. Participants were recruited between October 2015 and May 2022.

### 2.2. Data Collection

CSF samples (1 mL/participant) were obtained via lumbar puncture. Six LPA species (16:0, 18:0, 18:1, 18:2, 20:4, and 22:6), including positional isomers (sn-1 and sn-2), in the CSF samples were measured using liquid chromatography-tandem mass spectrometry (LC-MS/MS) [15]. The LC-MS/MS analysis was conducted using a Nexera system HPLC (Shimadzu Co., Kyoto, Japan) and LC-MS-8060 (Shimadzu). LC-MS-8060 includes a quantum triple-quadrupole mass spectrometer with a heated electrospray ionization source. Detailed procedures for measurement conditions and sample preparation were previously described [15], while C17:0 LPA was used as an internal standard for measuring LPA species in this study. The MRM transitions are listed in Table S1.

In this study, sn-1 and sn-2 LPA were measured separately, as they were well separated in several LPA and LPC species, as shown in Figure S2 (LPA) and Figure S3 (LPC). For statistical analysis, the CSF levels of undetectable LPAs were treated as zero for the primary analysis; however, this approach may introduce bias and should be interpreted with caution. Alternative approaches, such as imputation based on the limit of detection or censored data modeling, should be considered in future studies. In addition to the biochemical measurements, several clinical variables, including sex, age, and body weight, were recorded. The participants were asked to rate the average and maximum intensities of their NP and sleep status over the past week using an 11-point numerical rating scale (NRS; 0–10). Mental health status was assessed using the Hospital Anxiety and Depression Scale (HADS) and Pain Catastrophizing Scale (PCS), which measure catastrophic thoughts related to pain [8]. The HADS scores were separately analyzed as depression (HADS-D) and anxiety (HADS-A) subscales. LPC species were measured in parallel to confirm the analytical robustness of lysophospholipid profiling and enable an exploratory assessment of precursor–product relationships. Detailed information on concomitant medications was not systematically collected in this study, as the original study design primarily focused on biochemical profiling of CSF LPA species. Therefore, medication effects could not be incorporated into the statistical analyses. No missing clinical data were observed, except for undetectable LPA values, which were treated as zero as described above.

### 2.3. Statistical Analyses

Statistical analyses were designed to identify LPA molecular species associated with depressive symptoms and understand their relationships with pain-related clinical factors. All statistical analyses were conducted using JMP Pro version 16.1.0 (SAS Institute Inc., Cary, NC, USA). A two-sided significance level of *p* < 0.05 was used throughout.

Data normality was visually assessed using quantile–quantile (Q–Q) plots, and parametric statistical methods, including linear regression analyses, were applied accordingly. To identify explanatory variables associated with depressive symptoms, a correlation-based multivariate screening was conducted to evaluate the associations between individual LPA molecular species, clinical variables, and scores on the HADS-D. Variables indicating statistically significant associations with the HADS-D scores were retained for further analysis.

A bidirectional stepwise variable selection procedure was applied to the screened variables using predefined entry and removal criteria based on *p* values to mitigate potential multicollinearity and reduce model complexity. This two-stage variable selection strategy was adopted to limit overfitting and enhance the model interpretability. This approach was also intended to reduce potential selection bias. After confirming the absence of multicollinearity, the PCS scores, mean pain intensity, and LPA18:0 sn-1 were selected as explanatory variables in the final model. HADS-D scores were designated as the dependent variables, and multiple linear regression analyses were conducted.

Structural equation modeling (SEM) was subsequently conducted to further examine the interrelationships among depressive symptoms, LPA18:0 sn-1, and pain-related clinical variables indicated by regression analyses. Given the modest sample size, SEM was intentionally limited to a parsimonious model, including only observed variables that showed significant associations in prior regression analyses, to reduce overfitting and enhance model stability. The SEM parameters were estimated using the maximum likelihood method.

The overall model fit was evaluated using the chi-square test in combination with the comparative fit index (CFI), root mean square error of approximation (RMSEA), and standardized root mean square residual (SRMR). Model fit was deemed good with CFI ≥ 0.95 (acceptable ≥ 0.90), RMSEA ≤ 0.06 (acceptable ≤ 0.10), and SRMR < 0.08. When comparing competing models, the model with the smallest corrected Akaike Information Criterion (AICc) value was selected as the optimal model. Accordingly, SEM was used as an exploratory hypothesis-generating approach, instead of a confirmatory causal model. Given the exploratory nature of this study, no formal sample size calculation was performed. In addition, no formal correction for multiple comparisons was applied due to the exploratory nature of the analyses, and therefore the findings should be interpreted with caution. Accordingly, all statistical analyses should be considered hypothesis-generating, and the risk of type I error cannot be excluded.

## 3. Results

All analyses presented in this section should be interpreted as exploratory and hypothesis-generating. Table 1 shows the clinical characteristics of the participants. Six LPA species (16:0, 18:0, 18:1, 18:2, 20:4, and 22:6) were detected in the CSF of all the participants. Each LPA species is further classified into sn-1 and sn-2 subtypes according to the position of the acyl chain. Table 2 shows the results of the correlation analyses between HADS-D scores and individual LPA species.

There were no significant correlations between HADS-D scores and LPA species (LPA16:0, LPA18:1, and LPA20:4); these LPA species, however, have been reported to show linear associations with NP intensity. A significant correlation was observed between LPA18:0 and HADS-D scores (r = 0.380, *p* = 0.010), which has not been previously associated with pain intensity. Further analysis revealed that LPA18:0 sn-1 had a stronger correlation (r = 0.385, *p* = 0.009) than LPA18:0 sn-2 (r = 0.305, *p* = 0.041; Table 2). Focusing on the interrelationships between depressive symptoms, clinical variables, and LPA species, LPA18:0 levels showed no significant correlation with pain intensity, sleep disturbance, or PCS. In contrast, sleep disturbance was significantly correlated with the PCS (*p* = 0.040). HADS-D scores were designated as dependent variables, and multiple regression analyses were conducted. Several regression models are presented to demonstrate the robustness of the associations and the incremental contribution of key variables. The results indicate that depressive symptoms in patients with NP were explained by a model including average pain intensity, catastrophic thoughts about pain, and CSF LPA18:0 sn-1 levels (R^2^ = 0.386). Conversely, the explanatory power of PCS alone was lower (R^2^ = 0.188; Table 3).

**Figure 1:**
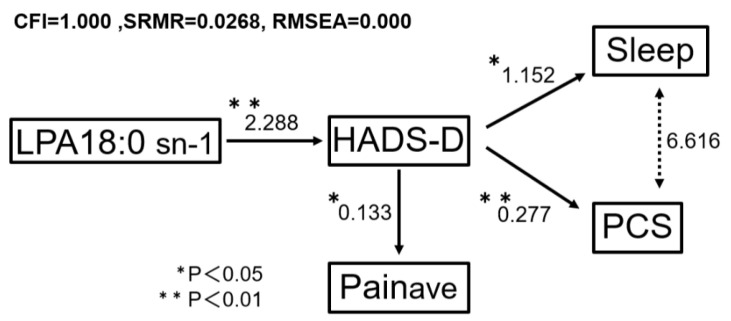
Structural equation model showing the relationships between depressive symptoms, pain-related factors, and LPA18:0 sn-1 in patients with neuropathic pain. Structural equation modeling was used to examine the relationships between depressive symptoms (HADS-D), LPA18:0 sn-1 levels in the cerebrospinal fluid, pain intensity, sleep disturbance, and pain catastrophizing. Directed paths represent statistical associations, while bidirectional arrows indicate covariances without an assumed directional relationship. Values represent standardized path coefficients. The SEM was constructed as an exploratory, parsimonious model to visualize the interrelationships among the observed variables rather than to establish causal inferences. Model fit indices indicated excellent fit (CFI = 1.000, SRMR = 0.0268, RMSEA = 0.000).

The inclusion of LPA18:0 sn-1 increased the explanatory power of the model compared to PCS alone. The relationships between PCS, sleep disturbance, mean pain intensity, and LPA18:0 sn-1 were found to be associated with HADS-D in the regression analysis and were modeled using SEM to create a path diagram, as shown in Figure 1. The model provides an excellent fit (CFI = 1.000, SRMR = 0.0268, RMSEA = 0.000; Figure 1), indicating that the proposed model adequately represented the observed data. Multiple factors influence depressive symptoms in patients with NP. Both analyses showed that LPA18:0 sn-1 was independently associated with HADS-D scores. In this cohort, no significant associations were found between the anxiety scores and any LPA molecular species. Exploratory analyses of cerebrospinal fluid LPC species and LPA/LPC ratios did not show any significant associations with depressive symptoms (Table S2).

## 4. Discussion

In this study, we identified CSF levels of LPA18:0 sn-1 as a molecular species associated with depressive symptoms in patients with NP. This association was independent of pain intensity or other pain-related clinical factors. Previous studies have primarily concentrated on the role of LPA in NP; in contrast, our findings highlight its clinical relevance to depressive distress.

### 4.1. The Significance of Evaluating Depression in Patients with Neuropathic Pain

The 2020 redefinition of pain describes it as “an unpleasant sensory and emotional experience associated with, or resembling that associated with, actual or potential tissue damage.” This definition emphasizes that pain perception includes sensory and emotional components, highlighting the importance of assessing emotional aspects of pain. In our previous study, we found that the CSF levels of certain LPA molecular species, including LPA16:0, LPA18:1, and LPA20:4, as well as total LPA, were linearly associated with NP severity [6]. The clinical significance of other LPA species, including LPA18:0 and LPA18:2, remains unclear, as they do not correlate with pain severity.

Among the various LPA species measured in this study, only LPA18:0 was significantly correlated with depressive symptoms. LPA18:0 sn-1 and LPA18:0 sn-2 were significantly correlated, with LPA18:0 sn-1 showing the strongest correlation (r = 0.385). Multivariate analysis identified LPA18:0 sn-1 as the only LPA species that independently contributed to depressive symptoms in conjunction with pain severity and catastrophic thoughts about pain. CSF LPA18:0 levels were not correlated with pain severity or other clinical variables. These findings indicate that LPA18:0, particularly the sn-1 isoform, may be considered a candidate biomarker associated with depressive symptoms in patients with NP; however, its clinical applicability remains to be established. Overall, these findings were integrated into a structural equation model to visualize the interrelationships between depressive symptoms, pain-related factors, and LPA18:0 sn-1. The SEM results should be interpreted as a conceptual framework rather than evidence of causality.

### 4.2. Potential Mechanisms Linking Depressive Symptoms in Neuropathic Pain to LPA

Various LPA species exist in human tissues and biological fluids, characterized by variations in fatty acid chain length and saturation. Each LPA species has distinct physiological functions in various tissues and organs. The turnover and clearance of LPA species in the central nervous system remain incompletely understood. Therefore, CSF LPA18:0 sn-1 levels may reflect dynamic pathophysiological processes rather than a stable steady-state marker. Potential mechanisms underlying increased CSF LPA18:0 sn-1 may include altered autotaxin activity, phospholipase-mediated lipid metabolism, and neuroinflammatory processes, although these remain to be elucidated. Recent research has revealed that LPA is involved in neuropsychological and neuropsychiatric disorders. Reportedly, the administration of LPA or LPA receptor 1 (LPAR1) agonists alleviates symptoms of depression and anxiety [12,16]. Furthermore, a tricyclic antidepressant, amitriptyline, directly binds to LPAR1 and activates downstream intracellular signaling, leading to antidepressant-like behaviors [17]. Consequently, LPA signaling through LPAR1 was considered the mechanism behind the non-monoaminergic antidepressant effects of tricyclic antidepressants.

Emerging evidence indicates that individual LPA molecular species have varying affinities and efficacies toward specific LPA receptors, resulting in potentially distinct downstream signaling profiles [18]. These findings suggest that alterations in the upstream fatty acid composition may selectively influence LPA-mediated signaling pathways rather than uniformly enhancing global LPA activity. To our knowledge, no studies have directly examined the relationship between LPA18:0 and depression. Consistent with this, postmortem analyses have reported increased levels of stearic acid (C18:0) in the brains of patients with mood disorders [19]. Since LPA18:0 is a bioactive derivative of stearic acid, these findings imply that stearate-derived lipid signaling pathways (LPA-LPA receptors) may be relevant to depressive symptomatology.

Previous studies have associated microglial activation with the pathophysiology of depression [20,21]. Increased microglial activation in the prefrontal cortex and hippocampus is positively correlated with depressive symptoms. Selective serotonin reuptake inhibitors and serotonin-norepinephrine reuptake inhibitors exert antidepressant effects, at least in part, by suppressing microglial activation [21]. These findings indicate that microglial activation and impaired neurogenesis are essential in the progression of depression. The association between LPA and microglial activation is well-established in the field of NP. LPA directly contributes to microglial activation and sensitization of spinal dorsal horn neurons, leading to neuronal hypersensitivity and pain amplification [22]. Furthermore, inhibiting the LPA-synthesizing enzyme ATX prevents microglial activation following nerve injury and alleviates NP [23].

The aim of this study was to investigate whether LPA molecular species are differentially associated with depressive symptoms in patients with NP, rather than generally focusing on LPA signaling. A distinct pattern was observed in the distribution of LPA molecular species associated with depressive symptoms in these patients. Metabolically, LPC is an upstream substrate for LPA production via ATX, while LPA reflects downstream bioactive signaling. Our findings indicate that LPA18:0 sn-1, but not LPC species, is associated with depressive symptoms, highlighting that affective distress in NP may be more closely related to specific LPA signaling processes than to global lysophospholipid availability. The LPA/LPC ratio was not the main endpoint of this study; however, it could provide complementary insights into lysophospholipid dynamics and warrants further investigation in future studies. Given LPA’s functional role in the central nervous system, these findings may be linked to microglial activation. Previous studies have indicated a relationship between LPA and the mental state via microglial activation [24].

Clinically, LPA18:0 sn-1 may offer additional information beyond the subjective psychological assessments. Since depressive symptoms are often under-recognized in patients with NP, an objective biomarker reflecting depressive distress may contribute to the identification of patients with increased depressive burden. These patients may benefit from closer psychiatric evaluations or early multidisciplinary interventions. Moreover, LPA18:0 sn-1 may serve as a stratification marker for future clinical trials targeting pain-associated depression. However, the reason for the stronger correlation between LPA18:0 sn-1 and LPA18:0 sn-2 remains unclear. There have been no studies that directly examined the relationship between LPA18:0 and depression at the positional isomer level. While sn-2 LPA species generally show stronger agonistic activity toward LPA receptors than sn-1 species, these findings imply that the observed association may not simply reflect the receptor potency. Since sn-1 lysophospholipids are predominantly generated by phospholipase A1, while sn-2 lysophospholipids are generated by phospholipase A2, the differential regulation of phospholipase activity may explain the preference for LPA18:0 sn-1 in relation to depressive symptoms. However, no previous studies have directly addressed the relationship between depression and phospholipase A1 activity, indicating the need for further investigation.

Building on previous work by our group, prior studies have highlighted the potential role of lysophospholipid signaling in neuropathic pain and neuropsychiatric conditions [25], supporting the broader relevance of LPA-related pathways.

### 4.3. Limitations

Several limitations of this study should be acknowledged. First, the association between CSF LPA18:0 sn-1 and HADS-D scores was not pre-specified as a primary endpoint and should therefore be interpreted as exploratory. This also raises the possibility of false-positive findings given the number of comparisons performed. Second, although structural equation modeling was used to explore interrelationships among depressive symptoms, pain-related clinical factors, and LPA species, the relatively small sample size may limit the stability and generalizability of the proposed model. Third, depressive symptoms were assessed using the Hospital Anxiety and Depression Scale depression subscale (HADS-D), which is a screening instrument reflecting symptom severity rather than a diagnostic tool for major depressive disorder. Fourth, cerebrospinal fluid sampling is an invasive procedure, which may limit the immediate routine clinical applicability of LPA18:0 sn-1 as a routine biomarker. Fifth, information on concomitant medications was not systematically collected in this study. This represents an important limitation, as several commonly prescribed drugs in neuropathic pain, including antidepressants (e.g., tricyclic antidepressants such as amitriptyline), anticonvulsants, and opioids, may influence LPA signaling pathways, including LPAR1-mediated mechanisms. Therefore, the potential confounding effects of pharmacological treatments on CSF LPA18:0 sn-1 levels cannot be ruled out and may have influenced the observed associations, and it was not possible to disentangle medication effects from the present results. Importantly, the present findings should be interpreted as reflecting real-world clinical conditions in which patients receive multimodal treatments, rather than isolated biological effects. From this perspective, the observed association may still be relevant as a pragmatic biomarker under routine clinical settings. Future studies incorporating detailed medication stratification or controlled designs will be essential to determine whether the observed association represents an independent biological relationship. Finally, the modest sample size of this study may limit statistical power; therefore, the findings should be validated in larger and more diverse populations.

## 5. Conclusions

The measurement of LPA18:0 sn-1 in the CSF may serve as a candidate biomarker pending further validation for the clinical assessment of depressive symptoms in patients with NP. Since depression contributes to treatment resistance, evaluating LPA18:0 sn-1 levels may offer new insights for managing and treating NP-associated depression. Future studies integrating longitudinal designs and multimodal biomarkers may help establish LPA18:0 sn-1 as a clinically meaningful indicator of affective burden in neuropathic pain.

## Figures and Tables

**Table 1:** Characteristics of patients with neuropathic pain.

Background, *n* = 48		
Age: mean (±SD)	67.89 (±11.32)	
Male: *n* (%)	22 (45.8%)	
BMI: mean (±SD)	22.37 (±4.88)	
Painmax: mean (±SD)	7.23 (±2.22)	
Painave: mean (±SD)	5.82 (±1.75)	
HADS-D: mean (±SD)	8.83 (±4.52)	
Sleep: mean (±SD)	5.21 (±3.36)	
PCS: mean (±SD)	32.13 (±11.39)	
Primary Disease	FBSS * (15)	Radiculopathy (3)
	Polyneuropathy (6)	Myelitis (3)
	Spinal cord injury (6)	Postherpetic neuralgia (2)
	Lumbar spinal canal stenosis (6)	Others (7)
**Others**	Myelomalacia, Phantom pain, Syringomyelia, OPLL	
	radicular pain invaded by lymphoma, After schwannoma surgery	

Abbreviations: SD, standard deviation; BMI, body mass index; HADS-D, Hospital Anxiety and Depression Scale–Depression subscale; PCS, Pain Catastrophizing Scale; FBSS, failed back surgery syndrome [* including adhesive arachnoiditis]; OPLL, ossification of posterior longitudinal ligament.

**Table 2:** Correlations between each LPA species and HADS-D. Pearson correlation analysis was conducted between each LPA molecular species and the Hospital Anxiety Depression Scale depression score. In addition, we showed a correlation between this study’s data and clinical factors that have been previously reported to be associated with depression during pain.

LPA Species	Mean (±SD) [ng/mL]	r	*p*-Value
LPA16:0	4.18 (±3.19)	0.269	0.074
sn-1	3.56 (±2.72)	0.265	0.078
sn-2	0.62 (±0.48)	0.289	0.055
LPA18:0	1.58 (±0.85)	0.38	*0.010* *
sn-1	1.37 (±0.74)	0.385	*0.009* *
sn-2	0.21 (±0.13)	0.305	*0.041* *
LPA18:1	2.05 (±1.67)	0.228	0.131
sn-1	1.78 (±1.46)	0.232	0.125
sn-2	0.28 (±0.21)	0.199	0.191
LPA18:2	1.04 (±2.70)	0.041	0.789
sn-1	0.89 (±2.32)	0.04	0.797
sn-2	0.15 (±0.38)	0.05	0.742
LPA20:4	0.70 (±1.23)	0.04	0.795
sn-1	0.60 (±1.06)	0.039	0.8
sn-2	0.09 (±0.17)	0.045	0.771
LPA22:6	0.22 (±0.19)	0.188	0.216
sn-1	0.19 (±0.17)	0.183	0.23
sn-2	0.03 (±0.02)	0.191	0.21
**Clinical data**		**r**	***p*** **-Value**
Pain average	5.82(±1.75)	0.3254	*0.043* *
Sleep score	5.21(±3.36)	0.3498	*0.029* *
PCS score	32.13(±11.49)	0.4315	*0.003* *

* *p* values < 0.05 were considered statistically significant. Abbreviations: LPA, lysophosphatidic Acid; SD, standard deviation; PCS, Pain Catastrophizing Scale.

**Table 3:** Multiple regression analysis identifying factors associated with depressive symptoms (HADS-D) in patients with neuropathic pain.

Target	Explanatory	β	R^2^	F Value	*p* Value
HADS-D	PCS	0.209	0.366	4.466	*0.006* *
	LPA18:0 sn-1	0.008			
	Painave	0.44			
	Sleep	−0.141			
HADS-D	PCS	0.2	0.386	6.716	*0.001* *
	LPA18:0 sn-1	−0.035			
	Painave	0.5947			
HADS-D	PCS	0.199	0.365	11.197	*0.0001* *
	LPA18:0 sn-1	1.391			
HADS-D	PCS	0.164	0.188	1.697	*0.003* *
HADS-D	LPA18:0 sn-1	2.128	0.148	7.48	*0.009* *

* *p* values < 0.05 were considered statistically significant. Abbreviations: R^2^, coefficient of determination; β, standardized partial regression coefficient; HADS-D, Hospital Anxiety and Depression Scale–depression subscale; LPA, lysophosphatidic acid; PCS, Pain Catastrophizing Scale.

## Data Availability

The datasets generated and analyzed during the current study are available from the corresponding author on reasonable request, subject to approval by the Institutional Review Board of The University of Tokyo and in accordance with applicable data protection regulations.

## Supplementary Materials

The following supporting information can be downloaded at: https://www.mdpi.com/article/10.3390/brainsci16060573/s1, Figure S1: Molecular structures; Figure S2: Separation of sn-1 and sn-2 LPA molecular species by LC-MS/MS; Figure S3: Separation of sn-1 and sn-2 LPC molecular species by LC-MS/MS; Table S1: MRM transitions used for LC-MS/MS quantification of LPA and LPC species; Table S2: Exploratory analyses of cerebrospinal fluid LPC species and LPA/LPC ratios in relation to depressive symptoms.

## References

[1] Baker D.L., Umstot E.S., Desiderio D.M., Tigyi G.J. (2000). Quantitative analysis of lysophosphatidic acid in human blood fractions. Ann. N. Y. Acad. Sci..

[2] Schober A., Siess W. (2012). Lysophosphatidic acid in atherosclerotic diseases. Br. J. Pharmacol..

[3] Mutoh T., Rivera R., Chun J. (2012). Insights into the pharmacological relevance of lysophospholipid receptors. Br. J. Pharmacol..

[4] Inoue M., Rashid M.H., Fujita R., Contos J.J., Chun J., Ueda H. (2004). Initiation of neuropathic pain requires lysophosphatidic acid receptor signaling. Nat. Med..

[5] Hayakawa K., Kurano M., Ohya J., Oichi T., Kano K., Nishikawa M., Uranbileg B., Kuwajima K., Sumitani M., Tanaka S. (2019). Lysophosphatidic acids and their substrate lysophospholipids in cerebrospinal fluid as objective biomarkers for evaluating the severity of lumbar spinal stenosis. Sci. Rep..

[6] Kuwajima K., Sumitani M., Kurano M., Kano K., Nishikawa M., Uranbileg B., Tsuchida R., Ogata T., Aoki J., Yatomi Y. (2018). Lysophosphatidic acid is associated with neuropathic pain intensity in humans: An exploratory study. PLoS ONE.

[7] Edamura T., Sumitani M., Hayakawa K., Inoue R., Abe H., Tsuchida R., Chikuda H., Ogata T., Kurano M., Aoki J. (2022). Different Profiles of the Triad of Lysophosphatidylcholine, Lysophosphatidic Acid, and Autotaxin in Patients with Neuropathic Pain Diseases: A Preliminary Observational Study. Pain Ther..

[8] Sullivan M.J.L., Bishop S.R., Pivik J. (1995). The Pain Catastrophizing Scale: Development and validation. Psychol. Assess..

[9] Meyer-Rosberg K., Kvarnström A., Kinnman E., Gordh T., Nordfors L.O., Kristofferson A. (2001). Peripheral neuropathic pain—A multidimensional burden for patients. Eur. J. Pain.

[10] Saita K., Sumitani M., Nikaido T., Sekiguchi M., Inoue R., Abe H., Konno S., Uchida K. (2021). Exponential correlations among neuropathic components, pain intensity, and catastrophic thoughts in patients with musculoskeletal pain disorder. Curr. Med. Res. Opin..

[11] McHorney C.A., Ware J.E., Raczek A.E. (1993). MOS 36-Item Short-Form Health Survey (SF-36): II. Psychometric and clinical tests of validity in measuring physical and mental health constructs. Med. Care.

[12] Moreno-Fernández R.D., Pérez-Martín M., Castilla-Ortega E., Rosell Del Valle C., García-Fernández M.I., Chun J., Estivill-Torrús G., Rodríguez de Fonseca F., Santín L.J., Pedraza C. (2017). maLPA1-null mice as an endophenotype of anxious depression. Transl. Psychiatry.

[13] Harrison S.M., Reavill C., Brown G., Brown J.T., Cluderay J.E., Crook B., Davies C.H., Dawson L.A., Grau E., Heidbreder C. (2003). LPA1 receptor-deficient mice have phenotypic changes observed in psychiatric disease. Mol. Cell. Neurosci..

[14] Treede R.D., Jensen T.S., Campbell J.N., Cruccu G., Dostrovsky J.O., Griffin J.W., Hansson P., Hughes R., Nurmikko T., Serra J. (2008). Neuropathic pain: Redefinition and a grading system for clinical and research purposes. Neurology.

[15] Okudaira M., Inoue A., Shuto A., Nakanaga K., Kano K., Makide K., Saigusa D., Tomioka Y., Aoki J. (2014). Separation and quantification of 2-acyl-1-lysophospholipids and 1-acyl-2-lysophospholipids in biological samples by LC-MS/MS. J. Lipid Res..

[16] Rosell-Valle C., Pedraza C., Manuel I., Moreno-Rodríguez M., Rodríguez-Puertas R., Castilla-Ortega E., Caramés J.M., Gómez Conde A.I., Zambrana-Infantes E., Ortega-Pinazo J. (2021). Chronic central modulation of LPA/LPA receptors-signaling pathway in the mouse brain regulates cognition, emotion, and hippocampal neurogenesis. Prog. Neuro-Psychopharmacol. Biol. Psychiatry.

[17] Kajitani N., Okada-Tsuchioka M., Inoue A., Miyano K., Masuda T., Boku S., Iwamoto K., Ohtsuki S., Uezono Y., Aoki J. (2024). G protein-biased LPAR1 agonism of prototypic antidepressants: Implication in the identification of novel therapeutic target for depression. Neuropsychopharmacology.

[18] Choi J.W., Herr D.R., Noguchi K., Yung Y.C., Lee C.W., Mutoh T., Lin M.E., Teo S.T., Park K.E., Mosley A.N. (2010). LPA receptors: Subtypes and biological actions. Annu. Rev. Pharmacol. Toxicol..

[19] McNamara R.K., Rider T., Jandacek R., Tso P. (2014). Abnormal fatty acid pattern in the superior temporal gyrus distinguishes bipolar disorder from major depression and schizophrenia and resembles multiple sclerosis. Psychiatry Res..

[20] Atanasova D., Lazarov N., Stoyanov D.S., Spassov R.H., Tonchev A.B., Tchekalarova J. (2021). Reduced neuroinflammation and enhanced neurogenesis following chronic agomelatine treatment in rats undergoing chronic constant light. Neuropharmacology.

[21] Kappelmann N., Lewis G., Dantzer R., Jones P.B., Khandaker G.M. (2018). Antidepressant activity of anti-cytokine treatment: A systematic review and meta-analysis of clinical trials of chronic inflammatory conditions. Mol. Psychiatry.

[22] Ueda H., Matsunaga H., Olaposi O.I., Nagai J. (2013). Lysophosphatidic acid: Chemical signature of neuropathic pain. Biochim. Biophys. Acta.

[23] Uranbileg B., Ito N., Kurano M., Kano K., Uchida K., Sumitani M., Aoki J., Yatomi Y. (2021). Inhibition of autotaxin activity ameliorates neuropathic pain derived from lumbar spinal canal stenosis. Sci. Rep..

[24] Nagata W., Koizumi A., Nakagawa K., Takahashi S., Gotoh M., Satoh Y., Ishizuka T. (2023). Treatment with lysophosphatidic acid prevents microglial activation and depression-like behaviours in a murine model of neuropsychiatric systemic lupus erythematosus. Clin. Exp. Immunol..

[25] Choi J.W., Chun J. (2013). Lysophospholipids and their receptors in the central nervous system. Biochim. Biophys. Acta.

