# Supplementary material for: Lysophosphatidic Acid 18:0 sn-1 in Cerebrospinal Fluid as a Potential Biomarker of Depressive Symptoms in Patients with Neuropathic Pain
Source: Brain Sci. 2026 May 28;16(6):573. doi: 10.3390/brainsci16060573 (PMC13296637; doi:10.3390/brainsci16060573)
Supplement: Supplementary file 1 [file brainsci-16-00573-s001.zip › brainsci-4322654-supplementary.pdf]

Supplemental Digital Content

Figure S1: Molecular structures of LPA and LPC.

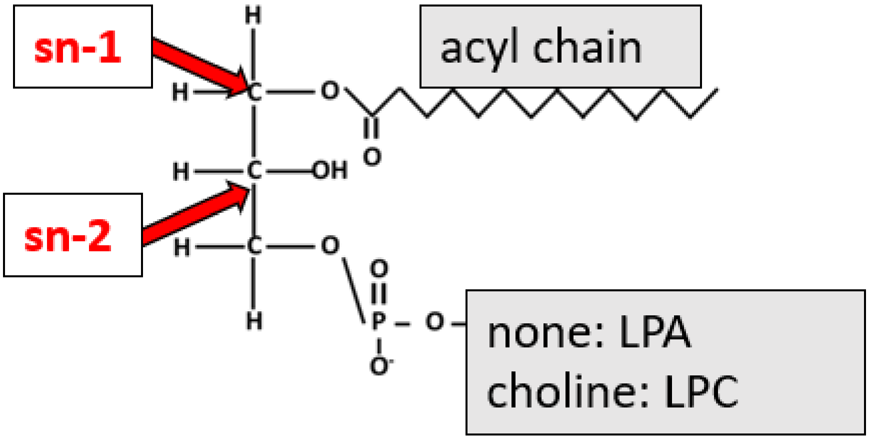

The positions of fatty acyl chains at the sn-1 or sn-2 position are shown.
Abbreviations: LPA, lysophosphatidic acid; LPC, lysophosphatidylcholine

Figure S2: Separation of sn-1 and sn-2 LPA molecular species by LC–MS/MS

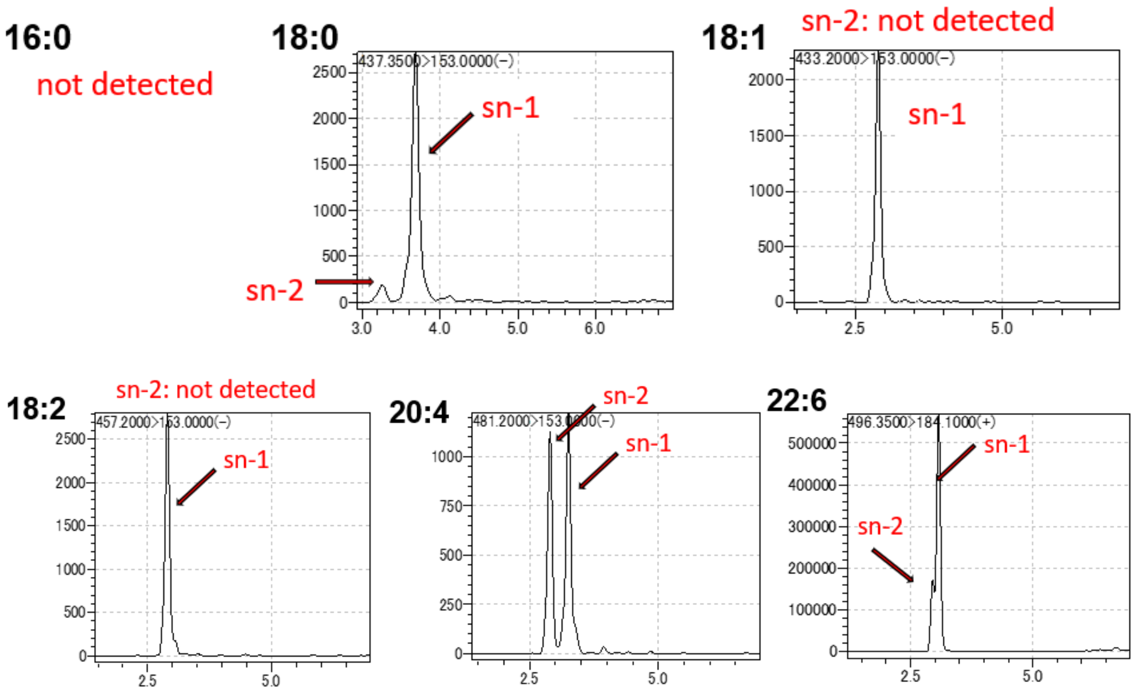

Representative chromatograms show clear separation of positional isomers.

Abbreviations: LPA, lysophosphatidic acid; LC–MS/MS, liquid chromatography–tandem mass spectrometry

Figure S3: Separation of sn-1 and sn-2 LPC molecular species by LC–MS/MS

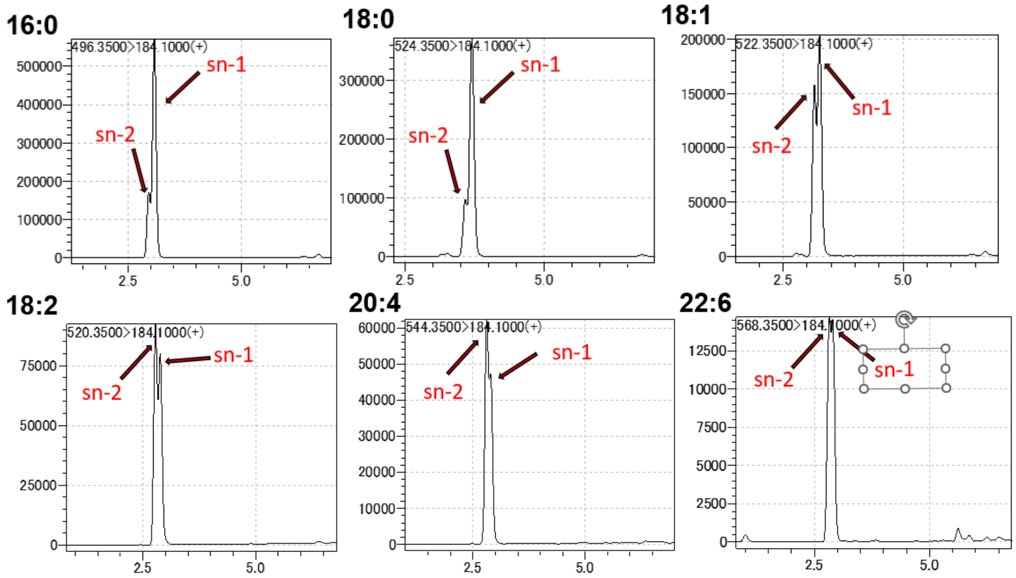

Abbreviations: LPC, lysophosphatidylcholine; LC–MS/MS, liquid chromatography–tandem mass spectrometry

Table S1: MRM transitions used for LC-MS/MS quantification of LPA and LPC species.

lipid species	m/z	lipid species	m/z
LPC 16:0	496.3500>184.1000	LPA 16:0	409.2500>184.1000
LPC 18:0	524.3500>184.1000	LPA 18:0	437.3500>153.0000
LPC 18:1	522.3500>184.1000	LPA 18:1	433.2000>153.0000
LPC 18:2	520.3500>184.1000	LPA 18:2	457.2000>153.0000
LPC 20:4	544.3500>184.1000	LPA 20:4	481.2000>153.0000
LPC 22:6	568.3500>184.1000	LPA 22:6	496.3500>184.1000

The table lists the precursor-to-product ion transitions (m/z) for each lysophospholipid species analyzed in cerebrospinal fluid, including the internal standards.

Abbreviations: MRM, multiple reaction monitoring; LPA, lysophosphatidic acid; LPC, lysophosphatidylcholine

Table S2: Exploratory analyses of cerebrospinal fluid LPC species and LPA/LPC ratios in relation to depressive symptoms

LPC species	mean(± SD) [ng/ml]	r	P-Value	LPA/LPC ratios	mean(± SD)	r	P-Value
LPC16:0	5.36(±17.79)	0.002	0.989	LPA/LPC16:0	2.44(±1.22)	0.182	0.232
sn-1	4.26(±14.64)	0.002	0.990	sn-1	2.95(±1.52)	0.149	0.330
sn-2	1.10(±3.15)	0.003	0.982	sn-2	1.26(±0.63)	0.248	0.100
LPC18:0	5.67(±13.41)	0.017	0.914	LPA/LPC18:0	0.55(±0.27)	0.145	0.343
sn-1	4.68(±10.88)	0.017	0.913	sn-1	0.57(±0.28)	0.145	0.343
sn-2	0.99(±2.54)	0.016	0.919	sn-2	0.48(±0.29)	0.070	0.649
LPC18:1	2.98(±5.62)	0.026	0.866	LPA/LPC18:1	1.19(±0.73)	-0.041	0.791
sn-1	1.70(±4.26)	0.010	0.948	sn-1	3.02(±2.35)	-0.102	0.504
sn-2	1.28(±1.39)	0.074	0.629	sn-2	0.26(±0.25)	-0.034	0.825
LPC18:2	0.85(±2.04)	0.029	0.852	LPA/LPC18:2	1.16(±0.76)	0.152	0.297
sn-1	0.43(±1.18)	0.011	0.943	sn-1	2.65(±1.83)	0.142	0.353
sn-2	0.42(±0.88)	0.052	0.733	sn-2	0.27(±0.22)	0.198	0.193
LPC20:4	0.46(±0.71)	0.058	0.703	LPA/LPC20:4	1.63(±0.92)	-0.039	0.797
sn-1	0.19(±0.35)	0.042	0.786	sn-1	5.72(±5.80)	-0.209	0.169
sn-2	0.27(±0.36)	0.074	0.629	sn-2	0.31(±0.21)	0.109	0.475
LPC22:6	0.26(±0.48)	0.058	0.706	LPA/LPC22:6	1.53(±1.14)	-0.056	0.716
sn-1	0.13(±0.30)	0.028	0.854	sn-1	4.02(±3.66)	-0.111	0.468
sn-2	0.13(±0.18)	0.105	0.494	sn-2	0.50(±1.15)	-0.074	0.631

Cerebrospinal fluid levels of individual lysophosphatidylcholine (LPC) molecular species and corresponding lysophosphatidic acid (LPA)/LPC ratios were analyzed to explore their potential relationships with depressive symptoms assessed using the Hospital Anxiety and Depression Scale depression subscale. LPC species were measured as upstream precursors of LPA to support the interpretation of LPA profiles and provide complementary information on lysophospholipid metabolism. Correlation analyses were conducted using Pearson correlation coefficients. These analyses were conducted exploratorily and were not designated as primary endpoints of the study.

Abbreviations: LPC, lysophosphatidylcholine; LPA, lysophosphatidic acid; SD, standard deviation
